# Benzhydryl phenyl sulfone

**DOI:** 10.1107/S1600536809050363

**Published:** 2009-11-28

**Authors:** Mahiuddin Baidya, Herbert Mayr, Peter Mayer

**Affiliations:** a Ludwig-Maximilians-Universität, Department, Butenandtstrasse 5–13, 81377 München, Germany

## Abstract

In the title compound, C_19_H_16_O_2_S, the sulfur-bound phenyl group is approximately parallel to one of the two phenyl rings of the benzhydryl group, making a dihedral angle of 12.53 (10)°, and forms a dihedral angle of 41.25 (9)° with the other phenyl ring. In the crystal, weak C—H⋯O inter­actions form a two-dimensional network propagating along the *bc* plane.

## Related literature

For background to the sulfone anion, see: da Silva Corrêa *et al.* (1968 ▶); Mayr *et al.* (2001 ▶, 2008 ▶). For a related structure, see: Li *et al.* (2005 ▶). For graph-set analysis of hydrogen-bond networks, see: Bernstein *et al.* (1995 ▶); Etter *et al.* (1990 ▶).
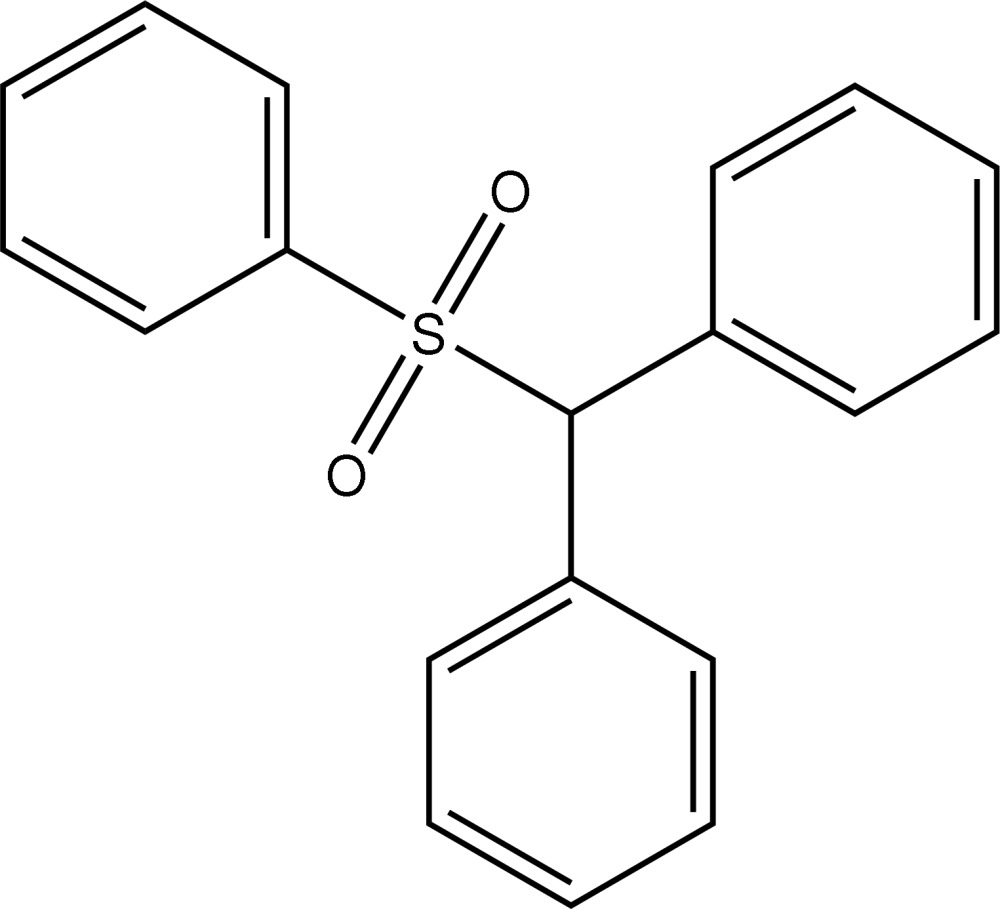


## Experimental

### 

#### Crystal data

C_19_H_16_O_2_S
*M* *_r_* = 308.40
Orthorhombic, 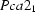

*a* = 16.3250 (4) Å
*b* = 5.7979 (1) Å
*c* = 16.4983 (4) Å
*V* = 1561.58 (6) Å^3^
*Z* = 4
Mo *K*α radiation
μ = 0.21 mm^−1^
*T* = 200 K
0.20 × 0.10 × 0.09 mm

#### Data collection

Nonius KappaCCD diffractometer
Absorption correction: none
11675 measured reflections
3499 independent reflections
3136 reflections with *I* > 2σ(*I*)
*R*_int_ = 0.027

#### Refinement

*R*[*F* ^2^ > 2σ(*F* ^2^)] = 0.031
*wR*(*F* ^2^) = 0.075
*S* = 1.04
3499 reflections
199 parameters
1 restraint
H-atom parameters constrained
Δρ_max_ = 0.14 e Å^−3^
Δρ_min_ = −0.28 e Å^−3^
Absolute structure: Flack (1983 ▶), 1646 Friedel pairs
Flack parameter: −0.03 (6)

### 

Data collection: *COLLECT* (Hooft, 2004 ▶); cell refinement: *SCALEPACK* (Otwinowski & Minor, 1997 ▶); data reduction: *DENZO* (Otwinowski & Minor, 1997 ▶) and *SCALEPACK*; program(s) used to solve structure: *SIR97* (Altomare *et al.*, 1999 ▶); program(s) used to refine structure: *SHELXL97* (Sheldrick, 2008 ▶); molecular graphics: *ORTEP-3* (Farrugia, 1997 ▶) and *Mercury* (Macrae *et al.*, 2006 ▶); software used to prepare material for publication: *PLATON* (Spek, 2009 ▶).

## Supplementary Material

Crystal structure: contains datablocks I, global. DOI: 10.1107/S1600536809050363/ds2011sup1.cif

Structure factors: contains datablocks I. DOI: 10.1107/S1600536809050363/ds2011Isup2.hkl

Additional supplementary materials:  crystallographic information; 3D view; checkCIF report

## Figures and Tables

**Table 1:** Hydrogen-bond geometry (Å, °)

*D*—H⋯*A*	*D*—H	H⋯*A*	*D*⋯*A*	*D*—H⋯*A*
C1—H1⋯O2^i^	1.00	2.46	3.449 (2)	171
C4—H4⋯O1^ii^	0.95	2.66	3.390 (2)	134
C7—H7⋯O2^i^	0.95	2.68	3.543 (2)	152

Symmetry codes: (i) 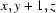; (ii) 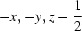.

**Fig. 1.**
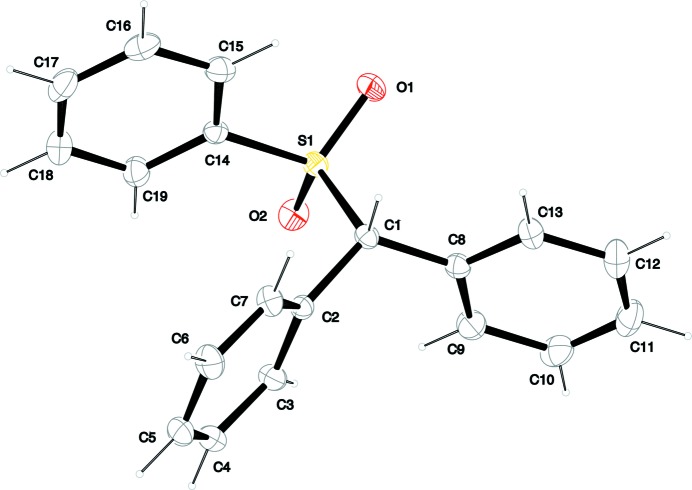
The molecular structure of the title compound, with atom labels and anisotropic displacement ellipsoids (drawn at 50% probability level) for non-H atoms.

**Fig. 2.**
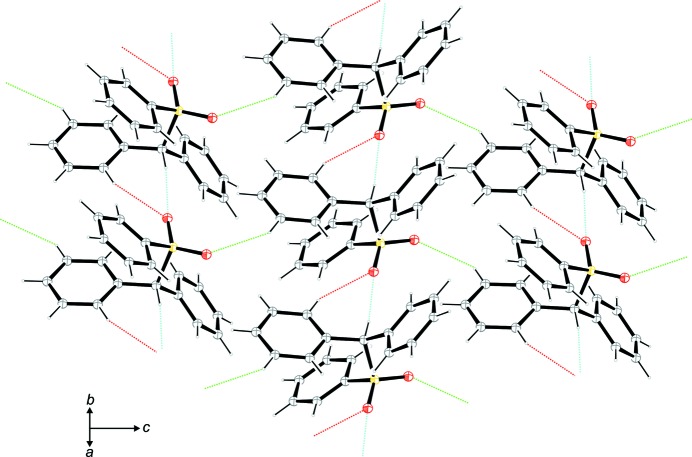
Weak intermolecular hydrogen bonds of the type C–H···O leading to a two-dimensional network that propagates along the bc plane (viewing direction approximately along [110]). Color scheme for dashed lines: blue: H1···O2 contacts, red: H7···O2 contacts, green: H4···O1 contacts.

## supplementary crystallographic information

### Comment

During our studies on the ambident reactivity of the phenylsulfinate anion we used diarylcarbenium ions (Ar_2_CH^+^) as reference electrophiles [Mayr *et al.* (2001, 2008)] and, hence, obtained the title compound from a reaction of sodium benzenesulfinate with benzhydryl chloride (Ph_2_CHCl) in dimethyl sulfoxide.

The asymmetric unit of the title compound contains one complete molecule, which is shown in Figure 1. The sulfur-bound phenyl group is approximately parallel to one of the two phenyl rings of the benzhydryl group with an dihedral angle of 12.53 (10)°. The other one forms a dihedral angle of 41.25 (9)° with the phenyl group bound to the sulfur atom.

Three weak C–H···O interactions are found (Table 1) which lead to the formation of a two-dimensional network that propagates along the *bc* plane (Fig. 2). Contacts of this type have been described for a structure of a related sulfone [Li *et al.* (2005)]. In terms of graph-set analysis [Bernstein *et al.* (1995), Etter *et al.* (1990)], the descriptors on the unitary level are C_1_^1^(4) for the H1···O2 interaction, C_1_^1^(6) for the H7···O2 interaction, and C_1_^1^(7) for the H4···O1 interaction.

### Experimental

Benzhydryl Phenyl Sulfone was obtained by heating a mixture of sodium benzenesulfinate (0.21 g, 1.3 mmol) and benzhydryl chloride (0.26 g, 1.3 mmol) in DMSO at 70 °C. After completion of the reaction (4 h), the reaction mixture was cooled to room temperature, diluted with water, and extracted with ethyl acetate. The organic phase was washed several times with water and dried (MgSO_4_). A viscous oil was obtained after evaporation of the solvent under reduced pressure that solidified on standing. After column chromatography (silica gel, isohexane/EtOAc = 9/1), benzhydryl phenyl sulfone was isolated as colorless solid (0.33 g, 82%). A small amount of the title compound was dissolved in ethyl acetate. The solvent was allowed to evaporate slowly at room temperature. After 2 days crystals had formed that were suitable for X-ray analysis. mp 189 °C (186–187 °C [da Silva Corrêa *et al.* (1968)]).

### Refinement

All H atoms were found in difference maps. C-bonded H atoms were positioned geometrically (C—H = 1.00 Å for aliphatic, 0.95 Å for aromatic H) and treated as riding on their parent atoms [*U*_iso_(H) = 1.2*U*_eq_(C)].

### Crystal data

**Table:**

C_19_H_16_O_2_S	*F*(000) = 648
*M**_r_* = 308.40	*D*_x_ = 1.312 (1) Mg m^−^^3^
Orthorhombic, *P**c**a*2_1_	Mo *K*α radiation, λ = 0.71073 Å
Hall symbol: P 2c -2ac	Cell parameters from 6504 reflections
*a* = 16.3250 (4) Å	θ = 3.1–27.5°
*b* = 5.7979 (1) Å	µ = 0.21 mm^−^^1^
*c* = 16.4983 (4) Å	*T* = 200 K
*V* = 1561.58 (6) Å^3^	Rod, colourless
*Z* = 4	0.20 × 0.10 × 0.09 mm

### Data collection

**Table:**

Nonius KappaCCD diffractometer	3136 reflections with *I* > 2σ(*I*)
Radiation source: rotating anode	*R*_int_ = 0.027
MONTEL, graded multilayered X-ray optics	θ_max_ = 27.5°, θ_min_ = 3.5°
Detector resolution: 9 pixels mm^-1^	*h* = −21→21
CCD; rotation images; thick slices, phi/ω–scan	*k* = −7→6
11675 measured reflections	*l* = −21→20
3499 independent reflections	

### Refinement

**Table:**

Refinement on *F*^2^	Secondary atom site location: difference Fourier map
Least-squares matrix: full	Hydrogen site location: inferred from neighbouring sites
*R*[*F*^2^ > 2σ(*F*^2^)] = 0.031	H-atom parameters constrained
*wR*(*F*^2^) = 0.075	*w* = 1/[σ^2^(*F*_o_^2^) + (0.0396*P*)^2^ + 0.2163*P*] where *P* = (*F*_o_^2^ + 2*F*_c_^2^)/3
*S* = 1.04	(Δ/σ)_max_ < 0.001
3499 reflections	Δρ_max_ = 0.14 e Å^−^^3^
199 parameters	Δρ_min_ = −0.28 e Å^−^^3^
1 restraint	Absolute structure: Flack (1983), 1646 Friedel pairs
Primary atom site location: structure-invariant direct methods	Flack parameter: −0.03 (6)

### Special details

**Table:**

Geometry. All e.s.d.'s (except the e.s.d. in the dihedral angle between two l.s. planes) are estimated using the full covariance matrix. The cell e.s.d.'s are taken into account individually in the estimation of e.s.d.'s in distances, angles and torsion angles; correlations between e.s.d.'s in cell parameters are only used when they are defined by crystal symmetry. An approximate (isotropic) treatment of cell e.s.d.'s is used for estimating e.s.d.'s involving l.s. planes.
Refinement. Refinement of *F*^2^ against ALL reflections. The weighted *R*-factor *wR* and goodness of fit *S* are based on *F*^2^, conventional *R*-factors *R* are based on *F*, with *F* set to zero for negative *F*^2^. The threshold expression of *F*^2^ > 2*σ*(*F*^2^) is used only for calculating *R*-factors(gt) *etc*. and is not relevant to the choice of reflections for refinement. *R*-factors based on *F*^2^ are statistically about twice as large as those based on *F*, and *R*-factors based on all data will be even larger.

### Fractional atomic coordinates and isotropic or equivalent isotropic displacement parameters (Å^2^)

**Table:**

	*x*	*y*	*z*	*U*_iso_*/*U*_eq_	
S1	0.06340 (2)	0.12898 (6)	0.26302 (3)	0.02887 (10)	
O1	0.08669 (8)	0.1945 (2)	0.34378 (7)	0.0408 (3)	
O2	0.03981 (7)	−0.10715 (19)	0.24877 (8)	0.0384 (3)	
C1	−0.01788 (10)	0.3229 (3)	0.23092 (10)	0.0276 (3)	
H1	0.0034	0.4820	0.2409	0.033*	
C2	−0.03155 (9)	0.3073 (3)	0.14009 (10)	0.0272 (3)	
C3	−0.06491 (11)	0.1160 (3)	0.10143 (11)	0.0347 (4)	
H3	−0.0816	−0.0139	0.1324	0.042*	
C4	−0.07391 (11)	0.1141 (3)	0.01781 (12)	0.0405 (4)	
H4	−0.0964	−0.0180	−0.0080	0.049*	
C5	−0.05082 (12)	0.2999 (4)	−0.02807 (12)	0.0428 (4)	
H5	−0.0576	0.2970	−0.0853	0.051*	
C6	−0.01740 (12)	0.4927 (4)	0.00955 (13)	0.0451 (5)	
H6	−0.0008	0.6218	−0.0218	0.054*	
C7	−0.00847 (11)	0.4955 (3)	0.09276 (11)	0.0363 (4)	
H7	0.0138	0.6283	0.1182	0.044*	
C8	−0.09145 (10)	0.2969 (3)	0.28610 (9)	0.0304 (4)	
C9	−0.14154 (12)	0.1021 (3)	0.28814 (12)	0.0431 (4)	
H9	−0.1290	−0.0274	0.2551	0.052*	
C10	−0.20970 (13)	0.0971 (4)	0.33832 (13)	0.0500 (5)	
H10	−0.2444	−0.0343	0.3383	0.060*	
C11	−0.22748 (13)	0.2805 (4)	0.38811 (13)	0.0531 (5)	
H11	−0.2740	0.2751	0.4227	0.064*	
C12	−0.17763 (14)	0.4719 (4)	0.38771 (14)	0.0547 (5)	
H12	−0.1895	0.5985	0.4223	0.066*	
C13	−0.10994 (12)	0.4802 (3)	0.33668 (11)	0.0406 (4)	
H13	−0.0760	0.6133	0.3365	0.049*	
C14	0.14411 (10)	0.2005 (3)	0.19613 (10)	0.0292 (3)	
C15	0.18653 (11)	0.4044 (3)	0.20890 (12)	0.0407 (4)	
H15	0.1739	0.5002	0.2539	0.049*	
C16	0.24784 (12)	0.4662 (3)	0.15468 (14)	0.0479 (5)	
H16	0.2775	0.6055	0.1623	0.057*	
C17	0.26549 (12)	0.3259 (4)	0.09010 (13)	0.0503 (5)	
H17	0.3075	0.3691	0.0532	0.060*	
C18	0.22315 (13)	0.1231 (4)	0.07798 (14)	0.0517 (5)	
H18	0.2364	0.0267	0.0333	0.062*	
C19	0.16122 (12)	0.0598 (3)	0.13093 (12)	0.0403 (4)	
H19	0.1311	−0.0782	0.1224	0.048*	

### Atomic displacement parameters (Å^2^)

**Table:**

	*U*^11^	*U*^22^	*U*^33^	*U*^12^	*U*^13^	*U*^23^
S1	0.03180 (18)	0.02898 (18)	0.02584 (18)	0.00027 (14)	−0.00044 (18)	0.00176 (17)
O1	0.0447 (7)	0.0530 (8)	0.0247 (6)	0.0025 (6)	−0.0042 (5)	0.0005 (5)
O2	0.0408 (6)	0.0273 (6)	0.0471 (9)	−0.0006 (4)	0.0029 (6)	0.0047 (5)
C1	0.0315 (8)	0.0251 (8)	0.0263 (8)	−0.0019 (6)	0.0000 (7)	−0.0014 (6)
C2	0.0257 (7)	0.0301 (8)	0.0258 (8)	0.0028 (6)	0.0008 (6)	0.0011 (6)
C3	0.0384 (9)	0.0357 (9)	0.0301 (9)	−0.0062 (7)	−0.0017 (7)	−0.0001 (7)
C4	0.0394 (9)	0.0507 (11)	0.0312 (10)	−0.0053 (8)	−0.0032 (8)	−0.0060 (8)
C5	0.0423 (10)	0.0597 (12)	0.0262 (9)	0.0046 (9)	−0.0005 (8)	0.0006 (8)
C6	0.0561 (13)	0.0471 (11)	0.0321 (9)	0.0014 (9)	0.0063 (9)	0.0098 (7)
C7	0.0440 (10)	0.0316 (9)	0.0334 (9)	−0.0002 (7)	0.0045 (7)	0.0013 (7)
C8	0.0327 (8)	0.0324 (8)	0.0261 (9)	0.0015 (6)	−0.0017 (6)	0.0028 (6)
C9	0.0433 (10)	0.0450 (11)	0.0410 (11)	−0.0093 (8)	0.0040 (8)	−0.0017 (8)
C10	0.0403 (10)	0.0638 (13)	0.0460 (12)	−0.0138 (9)	0.0040 (9)	0.0146 (10)
C11	0.0409 (10)	0.0738 (14)	0.0445 (12)	0.0098 (10)	0.0133 (9)	0.0159 (11)
C12	0.0575 (13)	0.0597 (13)	0.0470 (12)	0.0113 (10)	0.0174 (10)	−0.0022 (10)
C13	0.0465 (10)	0.0412 (10)	0.0340 (10)	0.0044 (8)	0.0048 (8)	−0.0049 (8)
C14	0.0270 (8)	0.0333 (8)	0.0273 (8)	0.0008 (6)	−0.0026 (7)	0.0016 (7)
C15	0.0384 (10)	0.0429 (10)	0.0409 (11)	−0.0064 (8)	−0.0044 (8)	−0.0039 (8)
C16	0.0340 (9)	0.0507 (11)	0.0589 (13)	−0.0111 (9)	−0.0065 (9)	0.0088 (10)
C17	0.0320 (9)	0.0683 (13)	0.0505 (12)	0.0027 (9)	0.0089 (9)	0.0144 (10)
C18	0.0466 (11)	0.0625 (13)	0.0459 (12)	0.0048 (9)	0.0128 (10)	−0.0074 (10)
C19	0.0402 (9)	0.0394 (9)	0.0414 (11)	0.0016 (8)	0.0045 (8)	−0.0056 (8)

### Geometric parameters (Å, °)

**Table:**

S1—O1	1.4366 (13)	C9—C10	1.387 (3)
S1—O2	1.4415 (12)	C9—H9	0.9500
S1—C14	1.7681 (17)	C10—C11	1.374 (3)
S1—C1	1.8180 (16)	C10—H10	0.9500
C1—C8	1.514 (2)	C11—C12	1.376 (3)
C1—C2	1.518 (2)	C11—H11	0.9500
C1—H1	1.0000	C12—C13	1.390 (3)
C2—C3	1.390 (2)	C12—H12	0.9500
C2—C7	1.394 (2)	C13—H13	0.9500
C3—C4	1.387 (3)	C14—C19	1.379 (2)
C3—H3	0.9500	C14—C15	1.386 (2)
C4—C5	1.369 (3)	C15—C16	1.389 (3)
C4—H4	0.9500	C15—H15	0.9500
C5—C6	1.390 (3)	C16—C17	1.371 (3)
C5—H5	0.9500	C16—H16	0.9500
C6—C7	1.381 (3)	C17—C18	1.378 (3)
C6—H6	0.9500	C17—H17	0.9500
C7—H7	0.9500	C18—C19	1.386 (3)
C8—C13	1.385 (2)	C18—H18	0.9500
C8—C9	1.395 (3)	C19—H19	0.9500
			
O1—S1—O2	118.23 (7)	C10—C9—C8	120.08 (19)
O1—S1—C14	108.64 (8)	C10—C9—H9	120.0
O2—S1—C14	108.67 (8)	C8—C9—H9	120.0
O1—S1—C1	107.45 (8)	C11—C10—C9	120.67 (19)
O2—S1—C1	110.16 (7)	C11—C10—H10	119.7
C14—S1—C1	102.53 (8)	C9—C10—H10	119.7
C8—C1—C2	118.10 (14)	C10—C11—C12	119.74 (19)
C8—C1—S1	110.02 (11)	C10—C11—H11	120.1
C2—C1—S1	110.99 (11)	C12—C11—H11	120.1
C8—C1—H1	105.6	C11—C12—C13	120.05 (19)
C2—C1—H1	105.6	C11—C12—H12	120.0
S1—C1—H1	105.6	C13—C12—H12	120.0
C3—C2—C7	118.25 (15)	C8—C13—C12	120.79 (18)
C3—C2—C1	123.92 (15)	C8—C13—H13	119.6
C7—C2—C1	117.83 (14)	C12—C13—H13	119.6
C4—C3—C2	120.27 (16)	C19—C14—C15	121.46 (17)
C4—C3—H3	119.9	C19—C14—S1	119.94 (13)
C2—C3—H3	119.9	C15—C14—S1	118.53 (13)
C5—C4—C3	120.94 (18)	C14—C15—C16	118.82 (18)
C5—C4—H4	119.5	C14—C15—H15	120.6
C3—C4—H4	119.5	C16—C15—H15	120.6
C4—C5—C6	119.59 (18)	C17—C16—C15	119.91 (18)
C4—C5—H5	120.2	C17—C16—H16	120.0
C6—C5—H5	120.2	C15—C16—H16	120.0
C7—C6—C5	119.67 (18)	C16—C17—C18	120.89 (19)
C7—C6—H6	120.2	C16—C17—H17	119.6
C5—C6—H6	120.2	C18—C17—H17	119.6
C6—C7—C2	121.27 (17)	C17—C18—C19	120.03 (19)
C6—C7—H7	119.4	C17—C18—H18	120.0
C2—C7—H7	119.4	C19—C18—H18	120.0
C13—C8—C9	118.64 (17)	C14—C19—C18	118.88 (18)
C13—C8—C1	117.33 (15)	C14—C19—H19	120.6
C9—C8—C1	124.03 (15)	C18—C19—H19	120.6
			
O1—S1—C1—C8	−61.76 (13)	C13—C8—C9—C10	1.9 (3)
O2—S1—C1—C8	68.31 (13)	C1—C8—C9—C10	−177.35 (17)
C14—S1—C1—C8	−176.16 (11)	C8—C9—C10—C11	−1.8 (3)
O1—S1—C1—C2	165.65 (11)	C9—C10—C11—C12	0.6 (3)
O2—S1—C1—C2	−64.28 (13)	C10—C11—C12—C13	0.5 (3)
C14—S1—C1—C2	51.25 (12)	C9—C8—C13—C12	−0.9 (3)
C8—C1—C2—C3	−59.0 (2)	C1—C8—C13—C12	178.46 (18)
S1—C1—C2—C3	69.33 (18)	C11—C12—C13—C8	−0.3 (3)
C8—C1—C2—C7	121.61 (17)	O1—S1—C14—C19	144.32 (14)
S1—C1—C2—C7	−110.03 (15)	O2—S1—C14—C19	14.45 (16)
C7—C2—C3—C4	0.6 (3)	C1—S1—C14—C19	−102.16 (15)
C1—C2—C3—C4	−178.76 (17)	O1—S1—C14—C15	−38.52 (16)
C2—C3—C4—C5	−0.5 (3)	O2—S1—C14—C15	−168.39 (13)
C3—C4—C5—C6	0.4 (3)	C1—S1—C14—C15	75.01 (15)
C4—C5—C6—C7	−0.5 (3)	C19—C14—C15—C16	−0.3 (3)
C5—C6—C7—C2	0.7 (3)	S1—C14—C15—C16	−177.45 (15)
C3—C2—C7—C6	−0.7 (3)	C14—C15—C16—C17	−0.2 (3)
C1—C2—C7—C6	178.68 (17)	C15—C16—C17—C18	0.0 (3)
C2—C1—C8—C13	−120.05 (16)	C16—C17—C18—C19	0.7 (3)
S1—C1—C8—C13	111.14 (15)	C15—C14—C19—C18	1.0 (3)
C2—C1—C8—C9	59.2 (2)	S1—C14—C19—C18	178.09 (15)
S1—C1—C8—C9	−69.59 (19)	C17—C18—C19—C14	−1.2 (3)

### Hydrogen-bond geometry (Å, °)

**Table:**

*D*—H···*A*	*D*—H	H···*A*	*D*···*A*	*D*—H···*A*
C1—H1···O2^i^	1.00	2.46	3.449 (2)	171
C4—H4···O1^ii^	0.95	2.66	3.390 (2)	134
C7—H7···O2^i^	0.95	2.68	3.543 (2)	152

Symmetry codes: (i) *x*, *y*+1, *z*; (ii) −*x*, −*y*, *z*−1/2.
